# O-GlcNAcylation is a mitochondrial-nuclear signal that regulates passive transport through the nuclear pore complex

**DOI:** 10.1016/j.jbc.2026.113343

**Published:** 2026-07-16

**Authors:** Talia Hart, Ashley Duke, Yifei Zhou, Alexander A. Soukas, Armen I. Yerevanian

**Affiliations:** 1 Center for Genomic Medicine and Diabetes Unit, Endocrine Division, Department of Medicine, Massachusetts General Hospital and Harvard Medical School, Boston, Massachusetts, USA; 2 Broad Institute of Harvard and MIT, Cambridge, Massachusetts, USA; 3 Program in Biological and Biomedical Sciences, Division of Medical Sciences, Harvard Medical School, Boston, Massachusetts, USA

**Keywords:** aging, biguanides, cancer cell biology, longevity, mitochondrial energetics, nuclear pore complex, O-GlcNAc, passive transport

## Abstract

The nuclear pore complex (NPC) is the single gateway between the nucleus and the cytoplasm, and in healthy cells there is a size threshold for passive diffusion across the NPC. In aging and disease, the NPC deteriorates, leading to promiscuous passive transport. We have previously showed that NPC protein expression is required for biguanide-induced lifespan extension, mTOR inhibition, and further that biguanide treatment leads to restriction of passive nuclear transport, but the underlying changes leading to this restriction were not identified. Here, we use fluorescent dextran transport and biochemical assays in HeLa cells to clarify the mechanism by which biguanide phenformin alters NPC permeability. We find phenformin treatment in HeLa cells leads to restricted passive nuclear transport in a dose and time-dependent manner. Multiple inhibitors of the mitochondrial electron transport chain also restrict passive nucleocytoplasmic transport. Critically, phenformin reduced expression of O-linked N-acetylglucosamine (O-GlcNAc) transferase, lowering global O-GlcNAcylation and locally decreasing O-GlcNAcylation of Nup98. O-GlcNAc transferase inhibition alone restricts passive transport, while increasing O-GlcNAcylation reverses phenformin’s effects. These results identify O-GlcNAc as a mitochondrial–nuclear signal and show that electron transport chain inhibition rapidly modulates nucleocytoplasmic transport *via* NPC post-translational modification in human cancer cells.

Nuclear pore complexes (NPCs) are large multiprotein channels embedded within the nuclear envelope that serve as the sole gatekeepers of nucleocytoplasmic traffic. Yeast, invertebrate nematode, and mammalian models indicate that as physiological aging occurs the NPC becomes “leakier”, allowing for promiscuous passive transport (1, 2, 3, 4, 5, 6, 7, 8, 9). Passive nuclear transport relies on the diffusion of molecules down their concentration gradient between the nucleus and cytoplasm. The molecular size limit for passive transport was originally reported to be 30 to 60 kD (kilodaltons), but more recent studies propose that the NPC is a soft barrier where larger molecules 60 to 230 kD may equilibrate across the pore on the scale of minutes to hours, respectively (10, 11, 12, 13). Some nuclear pore complex proteins or nucleoporins (Nups) contain intrinsically disordered phenylalanine-glycine domains (FG domains) that allow for both transient interactions with active transport receptors and form the permeability barrier within the channel. Although passive nuclear transport typically becomes more promiscuous with age, preservation of the NPC barrier is highly associated with longevity; several studies indicate that organisms with preserved NPC structure and function are longer lived (1, 9). What precisely contributes to stability in NPC function over time, and whether therapeutic targeting of passive transport is viable to promote healthy aging is an open area for investigation.

Our previous work revealed a connection between passive NPC transport and the oral hypoglycemic and geroprotective biguanide drug metformin (14). In addition to its well-established antidiabetic and cancer cell growth inhibition properties, metformin enhances the lifespan and healthspan of several model organisms (15, 16, 17, 18, 19, 20, 21, 22, 23, 24, 25, 26, 27, 28, 29, 30). We previously found that restricted nucleocytoplasmic transport is necessary for metformin to promote longevity in *C. elegans* and to block the growth of multiple cancer cell lines (14). Knockdown of several Nups including TPR, Nup98, Nup205, Nup214, and Nup85 prevents biguanide-mediated passive transport restriction in HeLa cells, suggesting that proper NPC composition and architecture are required for metformin activity (14). Highlighting an important role for mitochondria in this effect, mitochondrial inhibition by biguanides restricted passive nuclear transport, culminating in inactivation of the progeric and pro-growth kinase mTORC1. This signaling relay is necessary for metformin’s effects on cancer growth as well as lifespan in *C. elegans*, supporting the idea that governance of NPC function is required for metformin benefits in aging (14). Based upon these results, we hypothesized that NPC structure dynamics are directly affected by changes in mitochondrial energetics, and this mitochondria-NPC communication modulates passive transport as part of metformin’s pro-longevity and anti-cancer effects. How biguanide action is transduced to the NPC and what precise changes in NPC composition are mediated by biguanides is not known.

Recent studies have illustrated that post-translational modification (PTM) of Nups may be critical for controlling the speed of bidirectional transport (31, 32, 33, 34, 35). The O-linked N-acetyleglucosamine (O-GlcNAc) modification is a single N-acetylglucosamine bound to serine and threonine residues, acting much like phosphorylation to rapidly modulate protein function. Phenylalanine-glycine repeat rich nucleoporins (FG Nups) have long been known to be enriched for O-GlcNAc PTM, but how this affects transport has only recently been investigated. A recent study found that O-GlcNAc modification of inner channel FG Nups accelerates bidirectional passive transport of small inert proteins as well as active import and export (31). O-GlcNAc is generated in the cell through the hexosamine biosynthetic pathway (HBP) where it utilizes a variety of input metabolites, from glucose, glutamine, uridine and fatty acids to synthesize UDP-GlcNAc, the final substrate needed to produce O-GlcNAc (36). No matter the substrate, O-GlcNAc is added to and subsequently removed from proteins by a single pair of enzymes: O-GlcNAc transferase (OGT) and O-GlcNAc-ase (OGA), respectively (37). Altered levels of OGT and increased O-GlcNAc PTM of proteins has been observed in several types of cancers including breast cancer, prostate, leukemia, and hepatocellular carcinoma (38, 39, 40, 41, 42, 43). The HBP is known to be sensitive to its metabolic inputs including glucose, glutamine and acetyl-CoA, and ETC inhibition is associated with decreases in cytosolic glucose concentrations as greater quantities are shunted towards glycolysis for energy production (42). Abnormal O-GlcNAc levels have been associated with age-related diseases such as Alzheimer’s disease and diabetes (36).

Here, we show that treatment of human cells with the biguanide phenformin leads to a decrease in global O-GlcNAcylation levels, as well as significantly reduces O-GlcNAcylation of the FG Nup Nup98. Reduction in O-GlcNAc levels is accompanied by reduced expression of OGT, providing a plausible mechanism by which biguanides reduce O-GlcNAc modification of Nups. We find that direct inhibition of OGT is sufficient to restrict NPC passive transport, completely mimicking the effect of biguanides on the NPC. While increasing O-GlcNAcylation by inhibiting the enzyme that removes O-GlcNAc, OGA, reverses phenformin mediated effects on nuclear permeability. Inhibition of mitochondrial energetics with agents other than biguanides also restricts passive nuclear transport in an O-GlcNac-dependent manner, indicating this effect is likely to be mediated by mitochondrial-NPC signaling. By identifying O-GlcNac as a key Nup modification connecting alterations in mitochondrial activity to nuclear transport, our findings expand the known molecular consequences of biguanide action which could have relevance in both aging and cancer cell biology.

## Results

### Automated analysis of passive nuclear transport with the dextran uptake assay

**Figure 1:**
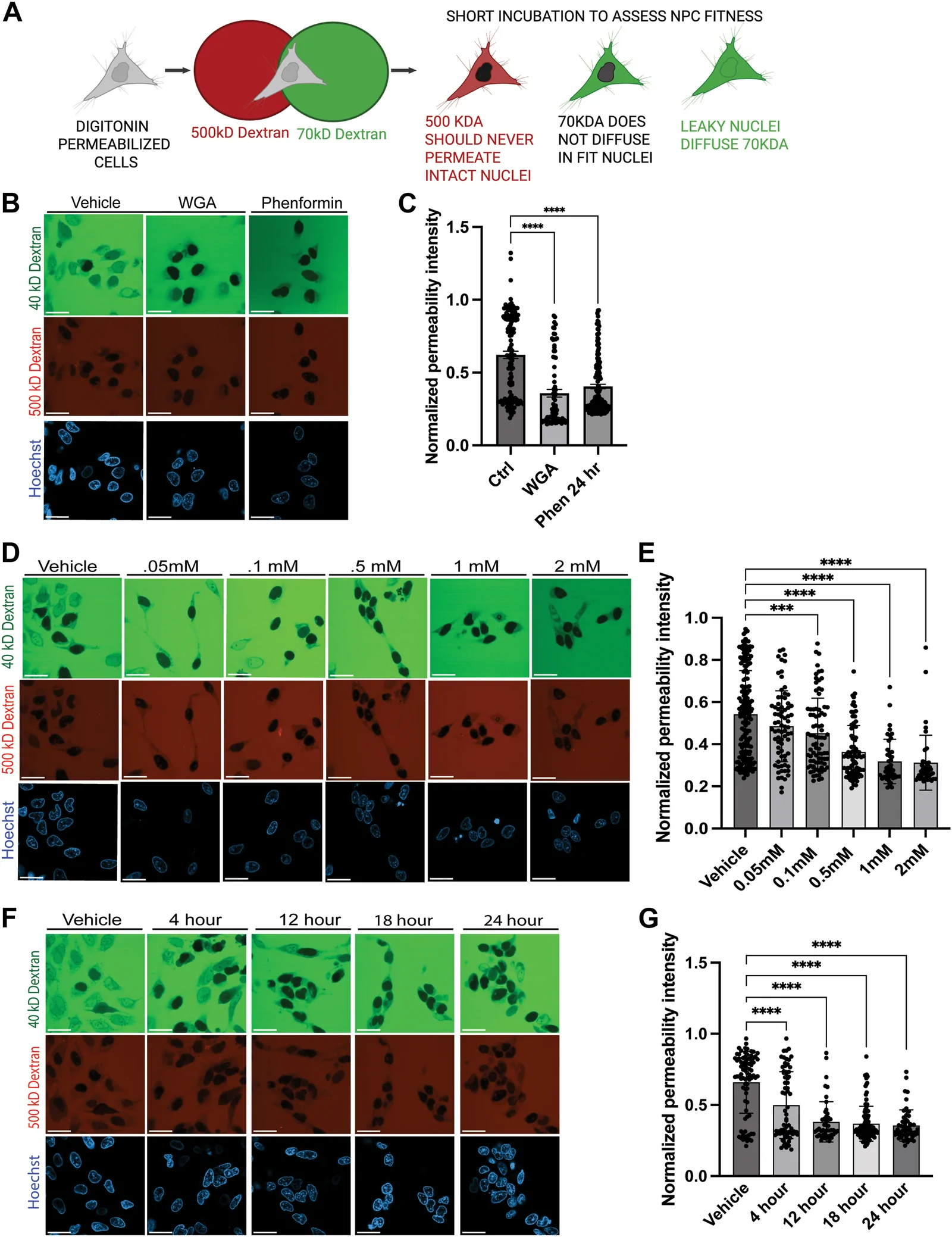
**Phenformin passive transport restriction is rapid and effective at low concentrations.***A,* workflow for dextran uptake assay. Cells are first permeabilized with digitonin and then subsequently incubated with dextrans conjugated to fluorescent tags and then assessed for alterations in nuclear transport. Representative images *B,* and quantification *C,* of nuclei in vehicle, germ agglutinin, or 1 mM phenformin treated HeLa cells analyzed with the dextran permeability assay using 40 kD FITC-Dextran and 500 kD TRITC-Dextrans co-stained with Hoechst, showing phenformin restricts passive transport of the 40kD dextran with as a positive control. Two independent replicates (n = 80–160 nuclei per condition). Representative images *D,* and quantification *E,* of HeLa cell nuclei in vehicle or dose range of phenformin from 0.05 mM to 2 mM for 24 h, analyzed with the dextran permeability assay, demonstrating an increase in restriction of the 40 kD FITC-Dextran with increased phenformin concentration. Two independent replicates (n = 50–245 nuclei per condition). Representative images *F,* and quantification *G,* of HeLa cell nuclei in vehicle or 1 mM phenformin for the labeled times between 4 and 24 h, analyzed with the dextran permeability assay-indicating a rapid restriction of the 40 kD FITC-Dextran that increases over time of exposure. Two independent replicates (n = 50–120 nuclei per condition). In each graph data points represent individual nuclei and bars indicate mean ± SD. Data analyzed using a one-way ANOVA followed by Dunnet’s multiple comparison test. ∗∗∗∗*p* < 0.0001. The scale bar represents 25 um. FITC, fluorescein isothiocyanat; TRITC, ttramethylrhodamine isothiocyanate.

We utilized the dextran permeability assay, a well-established *in vitro* method, to assess passive transport in cultured cells. Detergent-based permeabilization of the plasma membrane is followed by incubation with fluorophore-dextran conjugates, allowing for live assessment of passive transport (44). Treatment with 500 kD Tetramethylrhodamine isothiocyanate and 40 kD fluorophore-dextrans simultaneously allows for identification of cells with proper membrane permeabilization but intact nuclei, as a 500kD dextran will enter a permeabilized cell membrane but not an intact nucleus (Fig. 1*A*). As previously reported, cancer cells show enhanced permeability to passive cargoes (44, 45). We find that HeLa cells allow for rapid transport of 70 kD fluorescein isothiocyanate (FITC) dextrans (Fig. 1, *B* and *C*). In a previous study it was determined that biguanide treatment leads to restricted passive transport through the NPC (14). Phenformin reduces dextran permeability by 35% (Fig. 1, *B* and *C*). Wheat germ agglutinin, a lectin known to bind glycoproteins within the NPC and mechanically interfere with passive transport (46), was used as a positive control for restricted passive transport and reduced dextran permeability. The pancreatic carcinoma line Panc1 also exhibited a ∼50% decrease in nuclear permeability when treated with phenformin, indicating that phenformin effects are generalizable beyond HeLa cells (Fig. S1, *A* and *B*).

### Phenformin mediated passive transport restriction is rapid and effective at low concentrations

Biguanides such as phenformin and metformin have an extensive literature regarding cellular impacts on metabolism in numerous cell lines, at multiple concentrations and in both *in vitro* and *in vivo* settings. One previous study found 1 mM phenformin did not affect apoptosis markers in MeWo cells (47), while another had no significant effect on rates of programmed cell death at 100 μM, but at 1 mM phenformin had a modest ∼20% increase in apoptotic SH-SY5Y cells (48). In previous work (Wu *et al.*, 2016. *Cell*) (14), 1 mM Phenformin in transport and functional assays did not alter localization or apparent density of NPCs in HeLa cells as assessed by immunofluorescence and phenformin did not alter NPC component expression at the mRNA level in *C. elegans* and at 1 mM concentration in HEK293E and melanoma C8161 cells.

To better define the impact of phenformin action on nuclear permeability, we examined the effects of varying phenformin doses on passive nuclear restriction. To ensure that we examined effects of levels of phenformin on nuclear transport without pleiotropic effects on cell viability, we examined the impact of a range of drug concentrations within 24 h of treatment. At as low as 100 μM, we observed a dose-dependent increase passive nuclear restriction of 40 kD FITC-labeled dextran (Fig. 1, *D* and *E*). Despite reduced viability seen at the highest doses of phenformin, the dextran assay quantified living cells had a reduction of 42% in mean permeability intensity (Fig. 1, *D* and *E*). We find in the context of this transport assay that a 1 mM phenformin concentration maintains cell viability and function, while allowing for a broad enough dynamic range in passive transport values to find significant changes at this resolution of passive transport quantification.

As the rapidity of phenformin’s effect can also help to shed light on the underlying mechanism, we next asked how much phenformin exposure time is required to significantly restrict passive nuclear transport. We treated HeLa cells with a fixed 1 mM phenformin dose over timepoints ranging from 4 h to 24 h. At as early as 4 h of treatment phenformin treated HeLa cells exhibited a 25% decrease in nuclear permeability to 40 kD dextran *versus* control cells (Fig. 1, *F* and *G*). The drug effect plateaued at 12 h, with timepoints from 12 to 24 h maintaining a consistent, and a maximal restriction in nuclear dextran permeability of approximately 45% (Fig. 1, *F* and *G*), consistent with our dose-response analysis. These findings suggest that a rapid signaling cascade, possibly involving protein PTMs, alters NPC permeability in response to biguanide action even at low drug concentrations.

### Electron transport chain inhibition drives alterations in passive nucleocytoplasmic transport

**Figure 2:**
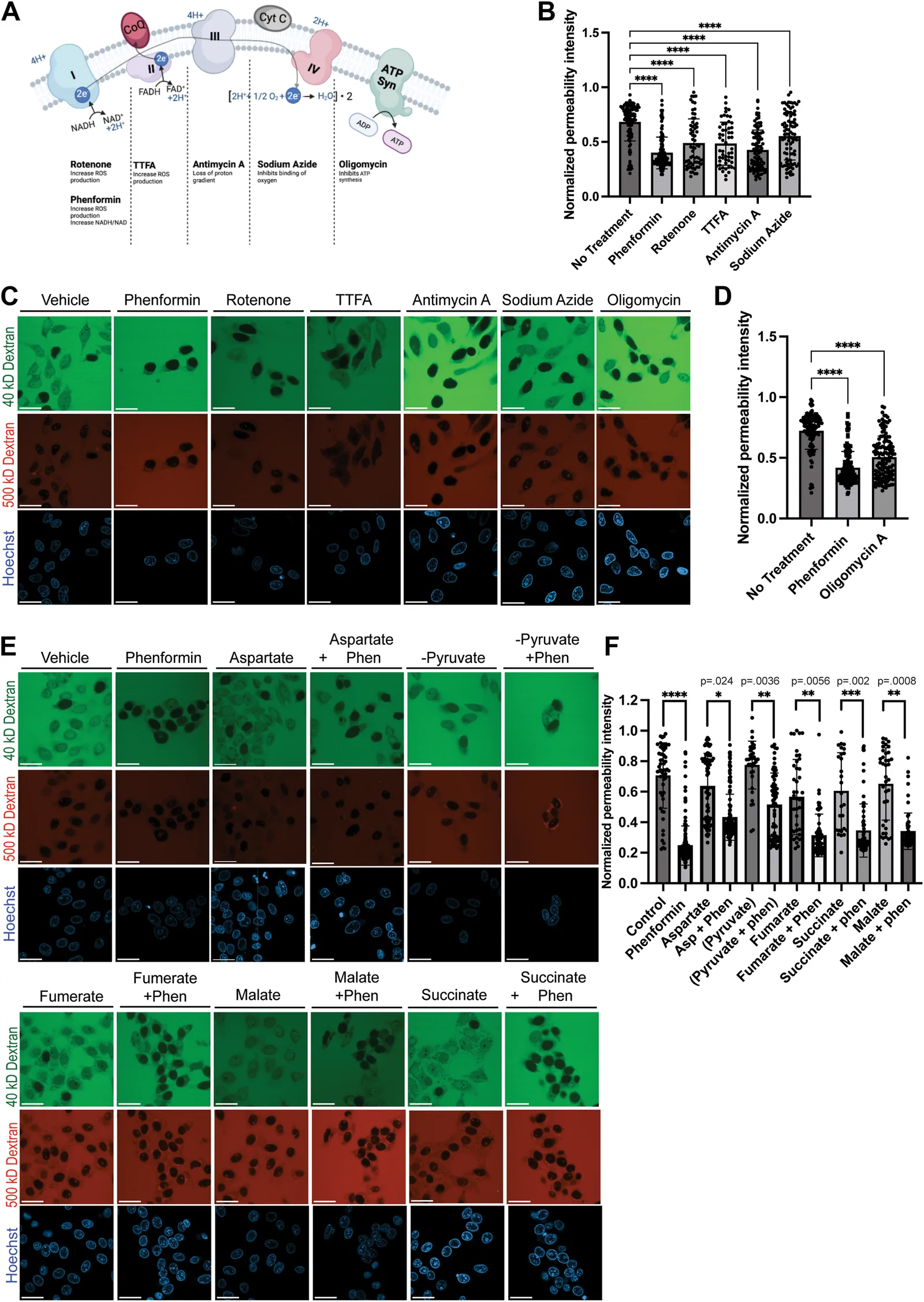
**Electron transport chain inhibition drives alterations in passive nucleocytoplasmic transport.***A,* overview of the ETC and site of action of complex-specific inhibitors. Nuclei from HeLa cells treated with vehicle, phenformin (1 mM), or ETC complex inhibitors rotenone (2.5 mM), TTFA (500 μM), antimycin A (1 mM), sodium azide (1 mM) and oligomycin (5 μg/ml) for 24 h, then permeabilized with digitonin and incubated with 40 kD FITC-Dextran, 500 kD TRITC-Dextran, and co-stained with Hoechst 33342. Representative images *C,* and quantification *B and D,* indicate that each of these ETC complex inhibitors significantly restricts passive transport of the 40kD dextran. Two independent replicates (*B*) and three (*D*) (n = 50–140 nuclei per condition). *E,*schematic of tricarboxylic acid cycle intermediates. *F,* quantitative analysis of nuclear permeability to 40 kD FITC dextran in permeabilized HeLa cells treated with vehicle or phenformin (1 mM) and mitochondrial metabolites aspartate (10 mM), fumarate (2.5 mM), malate (2 mM), and succinate (5 mM) or with pyruvate depleted media, for 24 h. Two independent experiments were conducted (n = 35–65 nuclei per condition). In each graph data points represent individual nuclei and bars indicate mean ± SD. Data analyzed using a one-way ANOVA followed by Dunnet’s multiple comparison test (*B* and *D*) and by a two-tailed Welch’s *t* test (*F*). ∗*p* < 0.05, ∗∗*p* < 0.01, ∗∗∗*p* < 0.001, ∗∗∗∗*p* < 0.0001, ns not significant. The scale bar represents 25um. ETC, electron transport chain; FITC, fluorescein isothiocyanat; TRITC, ttramethylrhodamine isothiocyanate.

**Figure 3:**
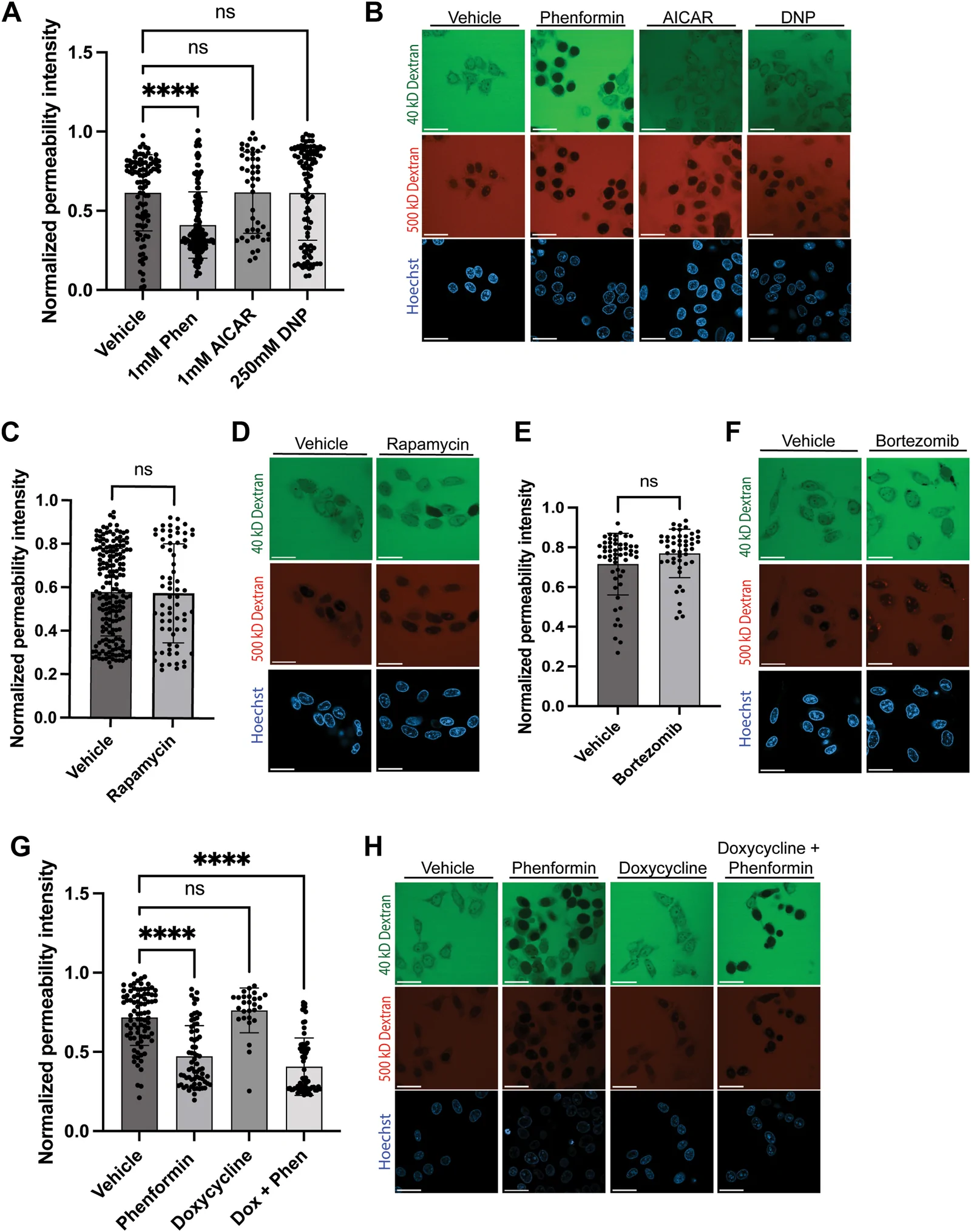
**Restricted passive transport is not activated through well characterized biguanide driven pathways.***A and B,* quantitative analysis of nuclear permeability to 40 kD FITC dextran in nuclei of permeabilized cells exposed to AICAR (1 mM for 24 h) and 2,4-dinitrophenol (250 mM for 24 h) compared to control (*A*) and representative images in (FB. n = 45–165 nuclei (*C* and *D*) quantitative analysis of nuclear permeability to 40 kD FITC dextran in nuclei of permeabilized cells exposed to100 nM rapamycin for 24 h compared to control (*C*) and representative images in (*D*). n = 65 to 185 nuclei (*E* and *F*) quantitative analysis of nuclear permeability to 40 kD FITC dextran in nuclei of permeabilized cells exposed to bortezomib (5 uM for 48 h) compared to control (*C*) and representative images in (*D*). n = 45 to 55 nuclei (*G* and *H*) quantitative analysis of nuclear permeability to 40 kD FITC dextran in nuclei of permeabilized cells exposed to doxycycline (20 μM for 48 h) and doxycycline with phenformin (1 mM for 24 h) compared to control and phenformin alone (*G*) and representative images in (*H*). n = 25 to 85 nuclei. The same nuclear dextran uptake assay was used in all assays in this figure in which cells permeabilized with digitonin were subsequently incubated for 20 min with 40 kD FITC-Dextran, 500 kD TRITC- Dextran, and Hoechst 33342 to illuminate effects of the indicated agents on passive nuclear transport. In each graph data points represent individual nuclei and bars indicate mean ± SD. Data analyzed using a one-way ANOVA followed by Dunnet’s multiple comparison test (*A*, *G*) and by a two-tailed Welch’s *t* test (*C*,*E*) ∗∗∗∗*p* < 0.0001, ns not significant. The scale bar represents 25um. FITC, fluorescein isothiocyanat; TRITC, ttramethylrhodamine isothiocyanate.

The precise mechanisms of biguanide action in diabetes, cancer growth inhibition, and aging remain elusive (21, 22, 23, 24), though recent *in vivo* studies suggest a mitochondrial mode of action (49). We hypothesized that phenformin restricts nucleocytoplasmic passive transport *via* the well-reported ability of biguanides to target and inhibit the function of mitochondrial Complex I in the electron transport chain (ETC) (24). Consistent with this hypothesis, treatment with several well-characterized, small molecule inhibitors of the ETC (Fig. 2*A*) was sufficient to restrict transport (Fig. 2, *B*–*D*). Phenformin and ETC Complex I inhibitor rotenone showed a quantitatively similar decrease in mean nuclear permeability compared to control. Similar results were obtained with inhibition of Complexes II through V (Fig. 2, *B*–*D*), indicating that restricted passive nuclear transport is a general response to inhibition of ETC activity. Biguanides and other ETC inhibitors also deplete levels of tricarboxylic acid (TCA) cycle intermediates (50, 51), and several prior studies have suggested that the major impact of ETC inhibition on cellular growth is to reduce the production of anabolic molecules such as aspartate and pyruvate (52, 53). Thus, we supplemented phenformin-treated cells with several TCA cycle intermediates and anabolic metabolites generated by mitochondria (Fig. 2, *E* and *F*) to determine if restoring TCA cycle flux or related metabolic intermediates restores permissive nuclear passive transport. Supplementation of cells with aspartate, fumarate, malate, and succinate did not impact the effect of phenformin on passive nucleocytoplasmic transport (Fig. 2, *E* and *F*). Reciprocally, media depleted of pyruvate failed to phenocopy the phenformin effect (Fig. 2, *E* and *F*). Thus, supraphysiologic levels of these metabolites are unable, either directly or indirectly, to reverse phenformin’s effects on nuclear restriction. 2,4-dinitrophenol (DNP), which uncouples the mitochondrial hydrogen gradient, had no discernable effect on passive nucleocytoplasmic transport (Fig. 3, *A* and *B*). While both ETC inhibition and DNP block ATP generation, ETC inhibition forbids further passage of electrons leading to a buildup of NADH and an increase in the ratio of reduced to oxidized NAD+, while DNP allows further passage of electrons and restoration of cellular NAD+ (54). From these findings we hypothesized that a cellular feature beyond ATP production *per se* acts downstream of ETC inhibition to effect changes in passive nuclear transport.

### Restricted passive transport is not activated through well characterized, downstream biguanide effector pathways

To investigate pathways downstream of ETC inhibition that could regulate NPC passive transport, we evaluated nuclear pore permeability following cell-treatments that impact known phenformin effector pathways. Phenformin and ETC inhibition are known to activate AMP-activated protein kinase (AMPK), which has been invoked as a key mediator of biguanide effects on metabolism and longevity (55). However, 5-aminoimidazole-4-carboxamide ribonucleotide, which stimulates AMPK activity directly, fails to restrict passive nuclear transport, indicating that activated AMPK is not part of the mito-NPC signaling relay (Fig. 3, *A* and *B*). Biguanide treatment has a well-known effect of inhibition of the mTOR growth pathway (23). Rapamycin, an antiproliferative drug and suppressor of mTOR, had no significant impact on passive nuclear transport (Fig. 3, *C* and *D*). This finding is in concert with our previously published data placing the ETC and nuclear pore complex genetically upstream of mTOR inhibition in metformin action (14). Next, we tested the proteasome inhibitor bortezomib, which inhibits cancer cell growth and induces apoptosis in breast cancer cells (56), to rule out the possibility that NPC restriction is not elicited through nonspecific toxic insults. Indeed, bortezomib had no effect on dextran nuclear restriction (Fig. 3, *E* and *F*). Finally, we investigated how mitochondrial protein synthesis inhibition, another downstream effect of phenformin treatment, impacts transport using a 48-h doxycycline treatment. This showed no alterations in passive nucleocytoplasmic transport, implying that the mito-nuclear signal does not stem from inhibited mitochondrial protein synthesis (Fig. 3, *G* and *H*). Collectively, these results suggest that mitochondrial communication to the NPC acts through ETC inhibition, but not through these well-described downstream mechanisms.

### Phenformin mediates downregulation of global and NPC O-GlcNAcylation

**Figure 4:**
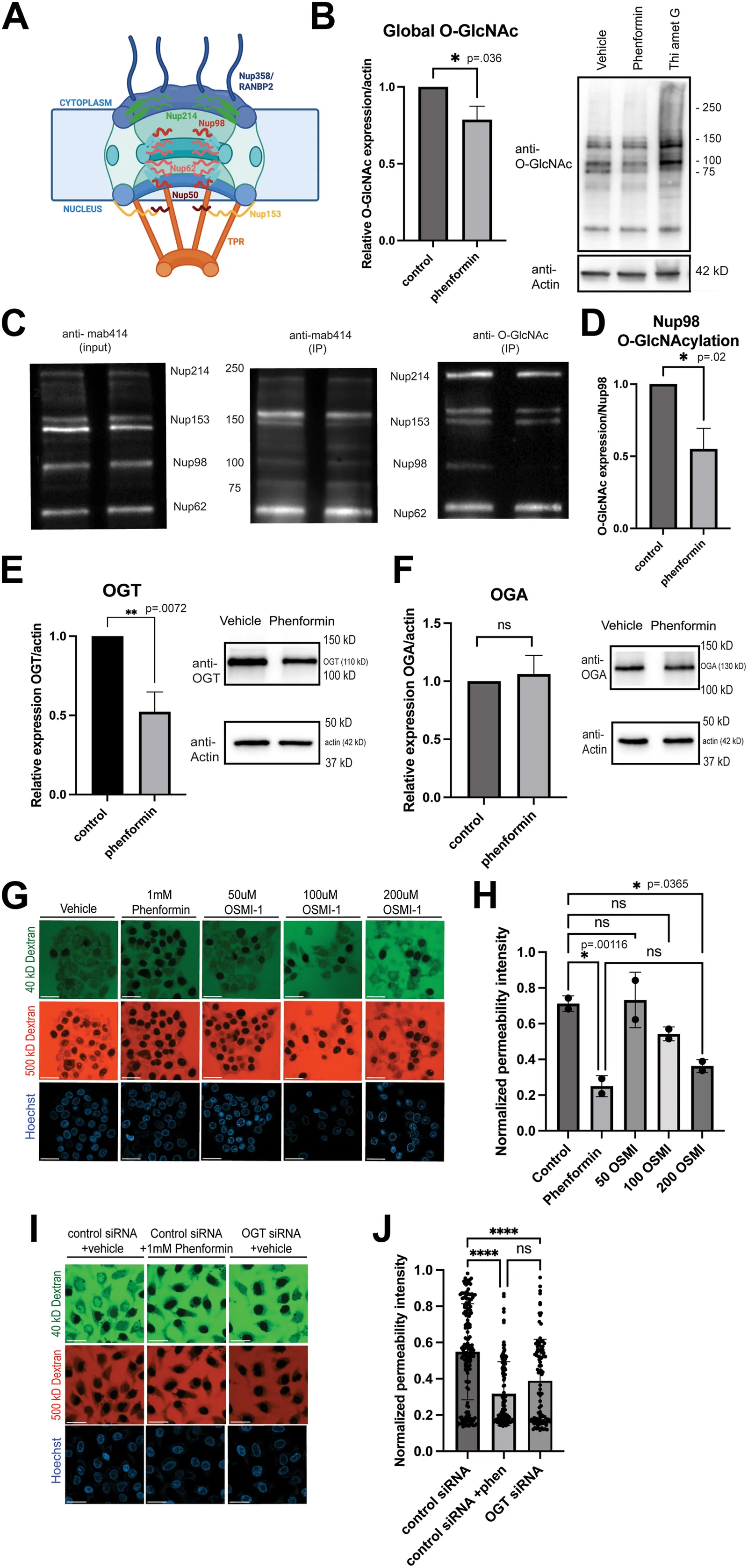
**Phenformin mediates down regulation of global and NPC O-GlcNAcylation through enzymatic regulation.***A,* Western blot representative image (*right*) and quantification (*left*) show total level of O-GlcNAc in HeLa cell lysates is significantly decrease with phenformin treatment. Protein extracts from HeLa cells treated with 1 mM phenformin or vehicle were probed for overall levels of O-GlcNAc modified proteins normalized to actin loading control. n = 6 biological replicates. *B,* simplified diagram of the Nuclear Pore Complex, highlighting key FG Nups immunoprecipitated with the mab414 antibody. Nup214 is on the cytoplasmic side and Nup153 within the basket extending into the nucleus. Nup98 and Nup62 are within the center channel. *C and D,* FG Nups immunoprecipitated by the mab414 antibody from vehicle and 1 mM phenformin treated HeLa cells and probed for levels of O-GlcNAc with mab414 acting as loading control indicates a decrease in O-GlcNAcylation of Nup98 with phenformin treatment. N = 4 biological replicates. Western blot probing for total levels of OGT (*E*) and OGA (*F*) with actin as a loading control indicate a significant decrease in OGT protein relative to Actin control in HeLa cells treated with 1 mM phenformin, with OGA levels unchanged. n = 3 biological replicates for both experiments. Representative images (*G*) and quantification (*H*) of HeLa cell nuclei treated with vehicle, 1 mM phenformin, 50,100, and 200 μM OGT inhibitor OSMI-1 for 24 h, analyzed with the dextran permeability assay demonstrating that inhibition of OGT alone is sufficient to induce passive transport restriction of the 40kD Dextran. Cells are incubated with a 40 kD FITC-Dextran, 500 kD TRITC-Dextran, and Hoechst 33342 co-strain. Two independent replicates (n = 160–300 nuclei per condition). Representative images (*I*) and quantification (*J*) of HeLa cell nuclei treated with negative control siRNA with and without phenformin treatment or OGT siRNA for 24 h. Two independent replicates (n = 100–165 nuclei per condition). In all graphs bars indicate mean ± SD, and dextran experiment data points represent individual nuclei (*H* and *J*). Data analyzed using a one-way ANOVA followed by Dunnet’s multiple comparison test (*H* and *J*) and by a two-tailed Welch’s *t* test (*A*, *D*, *E*, *F*) ∗*p* < 0.05, ∗∗*p* < 0.01, ∗∗∗∗*p* < 0.0001, ns not significant. Scale bar: 25um. OGT, O-GlcNac transferase; OGA, O-GlcNAc-ase; FITC, fluorescein isothiocyanat; TRITC, ttramethylrhodamine isothiocyanate.

One potential mechanism for phenformin mediated NPC restriction is through global changes in energetic capacity, where metabolite fluxes and redox ratios impact energy availability for transport dynamics at the nuclear pore. An alternative mechanism is a mito-nuclear signaling process driving acute structural changes to the nuclear pore complex in response to ETC inhibition. Restriction of passive nuclear transport in response to biguanides occurs within hours of treatment in mammalian cells, suggesting a rapid change in NPC dynamics. Nucleoporins bearing FG repeats form the NPC permeability barrier and are highly modified *via* PTMs that can regulate passive and active transport (32, 57) (Fig. 4*A*). Therefore, we hypothesized that PTM of accessible or exposed nucleoporins could be responsible for altered transport, rather than changes in transcription or translation of NPC components. The NPC is highly O-GlcNAclyated, leading us to investigate if phenformin effects O-GlcNAc levels of immunoprecipitated FG nucleoporins. We first measured the global level of O-GlcNAc modified proteins by immunoblotting total cellular lysates with and without phenformin treatment. O-GlcNAc in total lysates decreased by 25% compared to control, and was restored in the presence of thiamet G, a small molecule inhibitor of the O-GlcNAc removing enzyme OGA (Fig. 3*B*). Next, we immunoprecipitated the NPC with the monoclonal antibody mAb414, a well-established nuclear pore antibody which recognizes FG repeats (58), followed by Western blot probing for PTMs. Immunoblotting indicated successful enrichment of the expected NPC components at their appropriate sizes including Nup214, Nup153, Nup98, and Nup62, among others (Figs. 4*C* and S2*B*). From the mAb414 immunoprecipitation, a band corresponding to Nup98 had a ∼45% decrease in O-GlcNAcylation with phenformin treatment when compared to vehicle (Fig. 4*D*), while the other FG Nups did not show a significant decrease (Fig. S2, *B* and *C*). Nup98 is a crucial member of the NPC passive transport barrier, therefore this change in Nup98 O-GlcNAc levels could lead to altered Nup98 interactions with passively transported cargo (10, 31, 32, 33, 34, 35). We did not see a significant change in the global level of nucleoporins detected by mAb414 immunoblotting following phenformin treatment (Fig. S2*C*).

### OGT protein levels are significantly decreased in phenformin treated cells and inhibition of OGT is sufficient to restrict NPC passive transport

The global O-GlcNAc decrease led us to hypothesize that altered activity of OGT and OGA, which are the sole enyzmes that add and remove O-GlcNAc, respectively (37), could be regulated downstream of ETC inhibition leading to a decrease in O-GlcNAcylation. O-GlcNAc transferase (OGT) levels were significantly down regulated (Fig. 4*E*), while OGA levels are essentially unchanged with phenformin treatment (Fig. 4*F*). We then performed gene expression analysis of OGA and OGT in the presence of phenformin through RT-qPCR. Even though we see OGA total protein levels unchanged and OGT decreased with phenformin treatment (Fig. 4, *E* and *F*), we find that both OGT and OGA transcriptional expression is significantly upregulated (Fig. S2*E*). Because of this discrepancy between the mRNA and protein abundance of OGA and OGT with phenformin treatment, regulation of these enzymes is likely at the post-transcriptional level. Remarkably, treatment with 200 μM of OSMI-1, an inhibitor of OGT (59), was sufficient to induce a ∼50% decrease in passive nuclear permeability compared to vehicle treated cells, analogous to the effect of phenformin (Fig. 4, *G* and *H*). We next investigated if gene silencing through OGT siRNA treatment was sufficient to restrict passive transport through the NPC *in vitro*. In agreement with our inhibitor experiment we found that the cells treated with OGT siRNA for 24h had a significant decrease in import of the 70kD dextran when compared to the control siRNA, and comparable to 24 h 1 mM phenformin treatment (Fig. 4, *I*, *J* and S2*F*). This data collectively suggests a mechanism by which biguanides reduce O-GlcNAc levels *via* suppression of OGT protein expression, and further that OGT inhibition in isolation can act as a key driver in restricting NPC transport.

### Upregulated O-GlcNAcylation reverses phenformin mediated effects on nuclear permeability

**Figure 5:**
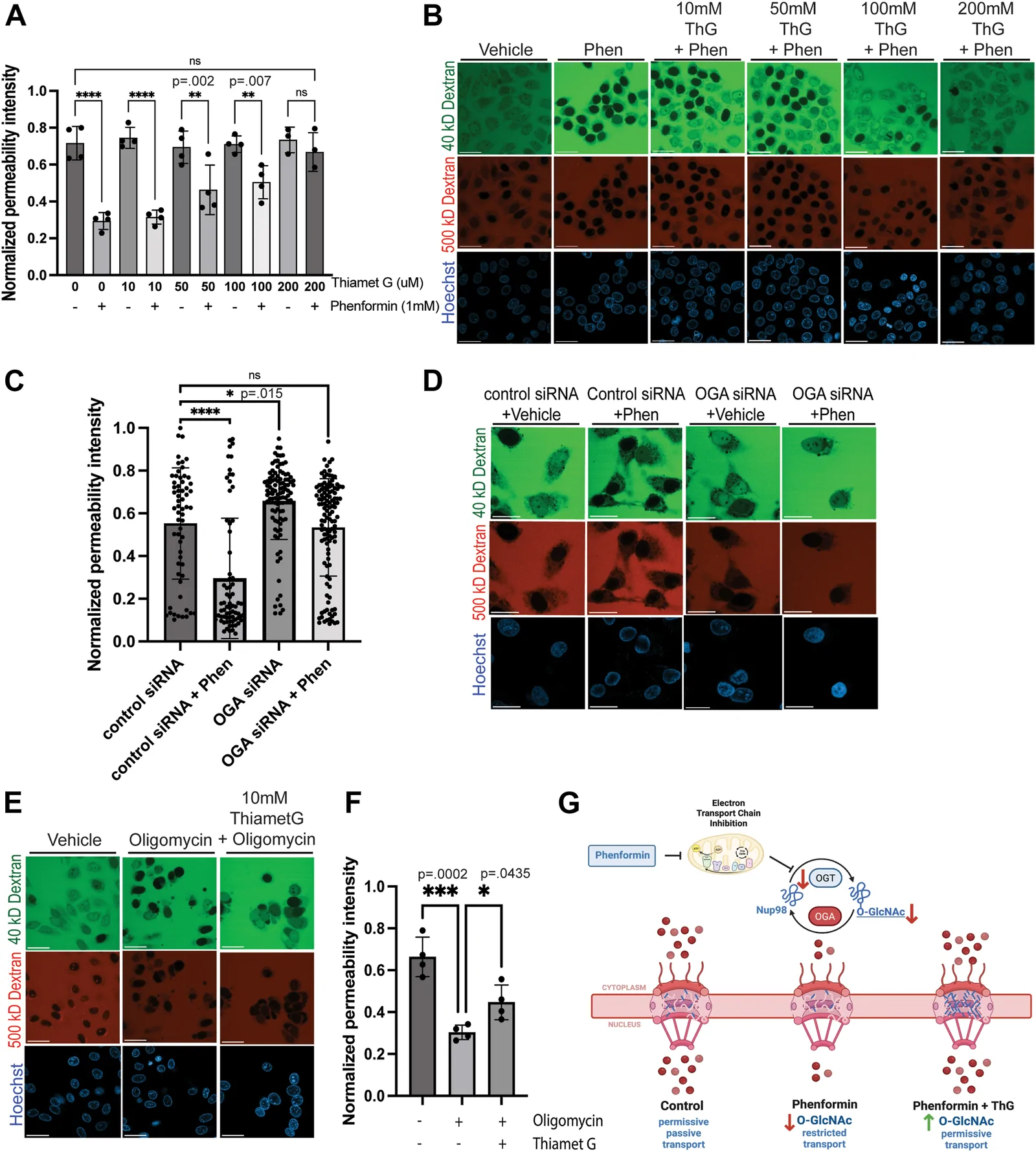
**Increased O-GlcNAcylation reverses phenformin mediated effects on nuclear permeability.** Quantification of 40kD dextran nuclear permeability intensity in HeLa cells with vehicle or phenformin (1 mM) treatment as well as increasing dosages of OGA inhibitor thiamet G (10 μm, 50 μM, 100 μM, 200 μM) as indicated (*A*) with representative images showed (*B*). Following drug treatment for 24 h, cells were permeabilized and incubated with 40 kD FITC-Dextran, 500 kD TRITC-Dextran, and co-stain Hoechst 33342. The mean permeability intensity increases with each higher dose of thiamet G, even in the presence of phenformin, leading to a complete rescue of leakiness at phenformin +200uM thG to the same permeability at vehicle control. This demonstrates that inhibition of OGA, which catalyzes O-GlcNAc removal from proteins, reverses phenformin restriction of passive nucleocytoplasmic transport. Four independent experiments were conducted (n = 80–180 nuclei per condition). Quantification (*C*) and representative images (*D*) of the dextran assay in HeLa cells treated with negative control siRNA with or without phenformin and OGA siRNA with or without phenformin demonstrating a similar response to Thiamet G treatment in that there was a rescue of the leakiness phenotype with OGA siRNA in the presence of phenformin. Quantification (*E*) and representative images (*F*) using the dextran assay protocol in HeLa cells treated with a combination of vehicle or 5 μg/ml oligomycin and with or without 100 μM thiamet G for 24 h, we see an increase in permeability intensity with thiamet G treatment in the restricted oligomycin treated cells, although not a complete rescue as vehicle and oligomycin + thiament G samples are still significantly different. In each graph data points represent individual nuclei and bars indicate mean ± SD. Data analyzed using a one-way ANOVA followed by Dunnet’s multiple comparison test (*A*, *C*, *F*) ∗*p* < 0.05, ∗∗*p* < 0.01, ∗∗∗*p* < 0.001, ∗∗∗∗*p* < 0.0001, ns not significant. The scale bar represents 10um. *G,* model summarizing findings. Biguanide mediated ETC inhibition, acting through the hexosamine biosynthetic pathway, promote a more stable NPC by reducing permissive passive transport. Expression of OGT is significantly decreased in phenformin treated cells, leading to a decrease in global O-GlcNAcylation levels, as well as O-GlcNAcylation of Nup98. We find inhibition OGT is sufficient to restrict NPC passive transport, and further upregulation of O-GlcNAcylation through thiament G treatment reverses phenformin mediated effects on nuclear permeability. ETC, electron transport chain; OGT, O-GlcNac transferase; OGA, O-GlcNAc-ase; FITC, fluorescein isothiocyanat; TRITC, ttramethylrhodamine isothiocyanate.

Our next aim was to examine the functional relationship between phenformin, nuclear restriction, and O-GlcNAc regulation. If decreased O-GlcNAcylation of the NPC is the mechanism through which biguanides act on the NPC, we expect that inhibition of OGA would mitigate phenformin-stimulated restriction of passive nucleocytoplasmic transport. To test this we performed a nuclear dextran assay with 1 mM phenformin and increasing amounts of the OGA inhibitor thiamet G with a range based on previously published data (10–200 μM) (31, 60, 61). Indeed, in a dose-dependent manner, thiamet G reverses phenformin’s ability to restrict nuclear passage of the 40 kD fluorophore-dextran conjugate(Fig. 5, *A* and *B*). Addition of 200 μM thiamet G to 1 mM phenformin treatment negates the biguanide effect, rendering nuclear permeability nearly identical to vehicle control (Fig. 5, *A* and *B*). To determine if post-transcriptional depletion of OGA replicates the result seen with the OGA inhibitor, we performed the dextran transport assay using HeLa cells treated with negative control siRNA with or without phenformin and OGA siRNA with or without phenformin. Indeed, we saw a similar response to Thiamet G treatment in that there was a rescue of the leakiness phenotype with OGA siRNA even in the presence of phenformin (Figs. 5, *C*, *D* and S2*F*).

This dramatic reversal led us to investigate if this is a general response to ETC inhibition. We observed a significant, albeit incomplete reversal of the oligomycin-induced restriction in nuclear permeability when cells were co-treated with 5 ug/ml oligomycin and 100 uM of thiamet G (Fig. 5, *E* and *F*). Collectively, this data supports that O-GlcNAc regulation is critical to elicit altered passive transport downstream of the biguanides action and ETC inhibition alike. These findings collectively link altered mitochondrial energetics to a rapid and consequential modulation of nucleocytoplasmic transport (Fig. 5*G*).

## Discussion

Using an established nuclear transport assay and biochemical analyses, we have identified a mechanism of cross talk between the mitochondria and nuclear pore complex in human cancer cells. Our results support a model whereby biguanide-mediated ETC inhibition, acting through the hexosamine biosynthetic pathway, reduces NPC passive transport. Specifically, the reduction in O-GlcNAc modification of FG Nups leads to less permissive passive transport, an effect previously tied to the health-promoting effects of biguanides (14). The elucidation of this signaling relay represents an advance in our understanding of cellular actions of biguanides, and further illuminates an unappreciated mito-nuclear signal that participates in age-related signaling (Fig. 5*G*). These findings highlight a layer of complexity to mitochondrial retrograde responses as well as cellular responses to altered mitochondrial bioenergetics.

Although our previous work found that the NPC transport was restricted upon biguanide treatment, and that this restriction was required for the pro-longevity and anti-cancer effects of the compounds (14), the signal driving this restriction remained unknown. NPC instability and increased permissive transport in aging are conserved features of age-related cellular decline across phylogeny (1, 2, 3, 4, 5, 6, 7, 8, 9), and FG Nup instability has been shown to be important to this effect (5, 6, 7, 8, 9). In our experiments, we did not see a change in total nucleoporin steady-state levels, and our previous work did not see a change in pore count with phenformin treatment in HeLa cells (14). Here, we show that permissive passive nuclear transport is malleable over a short time scale, a feature that would be unlikely if the sole mechanism of pore restriction was disruption of NPC stoichiometry.

Biguanides such as phenformin and metformin have an extensive literature regarding cellular impacts on metabolism in numerous cell lines, at multiple concentrations, and in both *in vitro* and *in vivo* settings. There is currently a debate on physiologically relevant drug treatment concentrations for the use of biguanides *in vitro* vs *in vivo*. Certain cellular compartments, such as the mitochondrial matrix, have higher concentrations of the drug (62), and studies find metformin to be highly concentrated in tissues of action vs the bloodstream-such as the intestine and brown adipose tissue (63, 64, 65)^,^.With this broad set of experimental models available, we focused on utilizing the smallest doses that trigger an experimentally viable dose-response effect in our paradigm of nuclear pore restriction. The goal of this approach is not to simulate the organ physiology of typical serum concentrations of biguanides but rather use them as a mechanism to trigger the types of energetic and ETC stress that activate a nuclear pore change. As more sensitive measures to study both NC transport and NPC O-GlcNAc levels emerge it will be possible to examine lower and more pharmaceutically relevant levels of biguanides for impact on these processes.

We found that beyond biguanides, inhibition of multiple ETC complexes ranging from Complex I through Complex V is sufficient to prompt restriction of passive nucleocytoplasmic transport. ETC inhibition with biguanides is known to decrease ATP levels, lower TCA activity, increase NADH/NAD+ ratios, and disrupt reactive oxygen Species production (higher redox) (24). Our experiments showed that replenishment of TCA metabolites alone was not sufficient to reverse the transport effect of phenformin, and depletion of ETC input factor pyruvate from media did not phenocopy phenformin mediated pore restriction, refuting a global energetic related mechanism. Lack of transport response to stimulation of AMPK agrees with our previous finding that biguanide-mediated NPC transport restriction is AMPK independent and unlikely related to ATP levels (14). Our finding that mTOR inhibition did not restrict passive transport is in concert with our previous work, placing the nuclear pore complex genetically upstream of mTOR inhibition in biguanide action (14). Another proposed mechanism for metformin’s glucose lowering effects is through Complex IV inhibition, leading to increased redox ratios, decreased pyruvate, and ultimately decreased glucogenesis (66). Complex IV inhibition did lead to pore restriction, but we did not find evidence of NPC restriction with pyruvate depletion, suggesting that mito-NPC effects are not driven by the precise mechanism laid out in that published work. Biguanide treatment leads to inhibited cancer cell growth, but inhibition of growth through bortezomib, a proteasome inhibitor and cancer treatment drug, did not phenocopy phenformin transport restriction. These experiments suggested that the global phenotype of ETC inhibition involving a low-energy and catabolic state was not the primary driver of the transport restriction, but that there is instead a direct form of communication between ETC and nuclear pore function.

This raised two potential mechanisms for how the ETC was having an impact. One through global changes in energetic capacity, metabolite fluxes, and redox ratios, and the other through mito-nuclear signaling. The hypothesis of NPC structural changes through signaling was particularly attractive because biguanides are known to impact downstream PTMs (23, 24). Therefore, the nuclear basket could serve as an excellent substrate for such global PTM rewiring. The significant enrichment of O-GlcNAc led us to measure levels in the context of biguanide-mediated ETC inhibition, and indeed we found a significant effect on Nup98 O-GlcNAc modifications. Our work does not refute the possibility that other nucleoporins are modified more subtly or in a more specific way than can be detected by methods used here. Nup98 is crucial to passive transport, with its intrinsically disordered FG-repeat domains contributing to the permeability barrier (10). Nup98 dysregulation is implicated in multiple aging-related diseases, including cancer, neurodegenerative diseases, and viral infections (3). Nup98 onco-fusion protein mutations are associated with poor outcomes in acute myeloid leukemia patients (67), and Nup98 is hijacked by SARS-CoV-2 ORF6 to block STAT1 nuclear translocation (68). How O-GlcNAcylation of Nup98 affects these diseases, and whether biguanide effects could intersect with these mechanisms could be an area of future research. These findings highlight a layer of mito-nuclear retrograde signaling complexity through specific metabolic signaling, with possible relevance in the context of stress, aging, and diseases.

In recent studies the HBP has emerged as an important energy sensor. The HBP requires input from multiple metabolic pathways, and its major output, UDP-GlcNAc, is linked to glycosylation events in key aging and oncogenic pathways (36, 38, 39, 40, 41, 42, 43). Given the links between glycosylation of the NPC and the HBP as an energy sensor, O-GlcNAc-based NPC modification could be a major way that cellular energetic status is read out as alterations in nuclear transport. Decreased O-GlcNAcylation of Nup98 with biguanides resonates with recent work by Yoo and Mitchison showing that decreased O-GlcNAcylation slows bidirectional nucleocytoplasmic transport (31). In contrast to these findings, one study demonstrated that mutation or knockdown of OGT in cells and aging *C. elegans* led to reduced O-GlcNAcylation and destabilization of nucleoporins, nucleoporin turnover and loss in aging, and more permissive passive nucleocytoplasmic transport (32). This difference could be due to chronic adaptation to loss of O-GlcNAcylation. Alternatively, total OGT loss could lead to pleiotropic impact on aging *versus* the blunted activity we report here with biguanides.

Even though we see OGA total protein levels unchanged and OGT decreased with phenformin treatment we find that both OGT and OGA transcriptional expression is significantly upregulated. In alignment with this finding, OGT and OGA have been found to co-regulate each other at the transcriptional level, leading to a current hypothesis in the field that this mutual regulation allows cells to maintain an optimum level of O-GlcNAcylation even with transient perturbations in nutrient flux, but can reach a tipping point and be disrupted in disease states (61, 69, 70, 71, 72, 73, 74). For example, one study compiling human cancer cell data sets found that OGA and OGT gene expression is highly correlated in numerous cancers at the transcriptional level (Qian *et al.* 2018) (70). Because of this discrepancy between the mRNA and protein abundance of OGA and OGT with phenformin treatment, regulation of these enzymes is likely at the post-transcriptional level. Indeed, several studies have found regulation of OGA and OGT activity through splicing, translation, and post translational modifications (75, 76, 77, 78, 79, 80). Overall, our current work demonstrates that the ratio of OGA to OGT protein abundance aligns with their activity to functionally impact O-GlcNAc levels and passive transport. How phenformin and general ETC inhibition leads to a net decrease in OGT protein levels remains an unresolved aspect of the mechanism and an area for future investigation.

This discrepancy raises questions regarding the most proximal mechanism connecting ETC inhibition with changes in O-GlcNAcylation. The HBP is known to be sensitive to its metabolic inputs, including glucose, glutamine, and acetyl-CoA. Because cell culture media provides potent enrichment of glucose and amino acids, this makes intracellular depletion of these substrates unlikely even in the context of moderate ETC inhibition. ETC inhibition may reduce O-GlcNAcylation activity through a decrease in UDP-GlcNAc production, and reductions in substrate are also known to negatively impact expression of OGT (61). Further research will be required to evaluate the extent of substrate sensitivity to this phenomenon under biguanide treatment. We propose that OGT expression is serving as a key regulatory step in governing O-GlcNAc levels at the nuclear pore complex. Studies have found increased uptake of glucose, higher OGT expression and hyperactivation of O-GlcNAc are signatures of cancer cells (36, 38, 39, 40, 41, 42, 43). Broad transcriptional changes with biguanide treatment and ETC inhibition more generally has been a well-documented phenomenon (23, 24). It will also be valuable to investigate how globally altering OGT introduces metabolic trade-offs for the cell. For example, one study found that glycolysis enzyme PGK1’s activity is enhanced by increased O-GlcNAc, promoting aerobic glycolysis and tumor growth (81).

Together, these findings support a model in which O-GlcNAc functions as a metabolic signal and effector at the NPC. This work highlights the advantage of maintaining NPC structural integrity and functionality in aging and suggests that modulating PTMs offers a beneficial intervention point. Our work uncovers a signaling axis between ETC activity and to reduced global O-GlcNAcylation, resulting in decreased nuclear permeability. This study underscores the importance of investigating the relatively understudied phenomenon of mitochondria–NPC crosstalk, particularly in the context of age-related disease. The possibility that NPC regulation contributes to the therapeutic benefits of biguanide and other treatments opens an exciting avenue for future research.

## Experimental procedures

### Experimental model and subject details

HeLa and PANC1 cells were purchased and verified through ATCC. Cells were grown in DMEM (Invitrogen) supplemented with 10% fetal bovine serum (Gemini Bio) and 1% penicillin-streptomycin (Life Technologies) in a 5% CO2 atmosphere at 37 °C.

### Reagents and antibodies used

Phenformin (Sigma Cat# PHR1573) was dissolved in water with the stock solutions at 1 M and volume added to treated cells was based on the indicated concentration. For dextran experiment treatments, 5-aminoimidazole-4-carboxamide ribonucleotide (LC Laboratories Cat#2627-69-2) was dissolved in water at 50 mM. Rotenone (Sigma Cat#R8875) and rapamycin (LC laboratories Cat#53123-88-9) were dissolved in ethanol to make 2.5 mM and 10 mM stock solutions, respectively. Tetramisole (Sigma, Cat#T1512), Oligomycin (Sigma Cat#495455), Antimycin A (Sigma Cat#A8674), Sodium Azide (Sigma Cat#S8032), OSMI-1 (Abcam Cat# ab235455), and Wheat germ agglutinin from Fisher Scientific (Cat#NC0082113) were used as cell treatments. Pyruvate, aspartate, malate, succinate, DNP, Bortezomib, Doxycycline (Cat# 24390-14-5) Thiamet G (Cat#SML0244) were obtained from Sigma. For western blotting, primary antibodies included O-GlcNAc RL2 (ab2739), OGT(D1D8Q) (Cell Signaling Cat#24083S), OGA (Abcam Cat# ab124807), actin (Cell Signaling Technologies Cat# [AC-40] ab11003) and mab414 (Biolegend Cat#902902). For immunostaining, secondary antibodies Goat anti-rabbit HRP conjugate (Thermo Fisher Scientific Cat# G21234) or Goat anti-mouse HRP conjugate (Thermo Fisher Scientific Cat#G21040) were used. Immunoprecipitation was performed using Affi-Prep Protein A magnetic beads (Biorad Cat#156-0006).

### Nuclear permeability dextran assay

HeLa cells were plated with 3 × 105 cells per well onto sterilized coverslips in the base of 6-well plates 24 h before drug treatment. After indicated hours of drug treatment, cell membranes were permeabilized with 40 mg/ml digitonin in freshly made transport buffer (TB) containing 20 mM Hepes, pH 7.3, 110 mM potassium acetate, 5 mM sodium acetate, 2 mM magnesium acetate, 0.5 mM EGTA, 2 mM DTT and protease inhibitor cocktail (Sigma Cat#11836170001) at room temperature. Digitonin (Sigma Cat#11024-24-1) permeabilization times were optimized by using trypan blue (Gibco Cat# 15250061) staining in HeLa cells (10 min) to efficiently permeabilize plasma membranes while keeping the nuclear envelope intact as indicated by full exclusion of FITC-Dextran 500000-Conjugate (Sigma 52194-1G). Immediately after semi-permeabilization, cells were rinsed twice with TB and incubated with fluorescent labeled dextran mixtures at the concentration of 2 mg/ml of each dextran for 20 min, including FITC-Dextran 40,000-Conjugate (Sigma FD40S-1G) mixed with TRITC-Dextran average molecular weight 500,000. Then, coverslips were inverted onto a 15 ml drop of Antifade mounting medium made with 1X PBS, 90% glycerol, and 2% n-propyl gallate (Sigma) and immediately imaged. Images were taken from randomly distributed fields of the coverslips using a Zeiss Airyscan confocal microscope outfitted with a standard GFP filter set or mCherry filter set at a 40X oil immersion objective. The fluorescence intensities of intranuclear area and extranuclear background for each nucleus were measured using CellProfiler, and the intensity ratio of intranuclear and extranuclear levels was calculated to index nuclear permeability.

### siRNA treatment

For dextran assay experiments using siRNA treatment, HeLa cells were plated with 3 × 105 cells per well onto sterilized coverslips in the base of 6-well plates. When cells reached approximately 70% confluency, they were treated with the indicated siRNA using the protocol for lipofectamine 3000 (Thermo Fisher Scientific, Cat #L3000008) modified for the use of siRNA as described in the manufacturer’s protocol. This protocol was performed using manufacturer validated siRNAs. This protocol was optimized for lipofectamine and siRNA concentration as well as incubation period to achieve knockdown as measured by qPCR. In brief, a working concentration of 5uM siRNA (silencer select siRNA, Thermo Fisher Scientific sequences) was combined with warmed opti-MEM media diluted for a final concentration of 37.5 nM OGT siRNA (Cat # 4390824, assay ID s16093, proprietary sequence) and corresponding control siRNA (Cat #4390843, proprietary sequence) and 10 nM for OGA siRNA(Cat# 4392420, assay ID s21065, proprietary sequence) and corresponding control in each well of the hela cell 6-well plates. Separately the lipofectamine 3000 reagent was suspended in warmed optiMEM media. Then both the siRNA and lipofectamine 3000 solutions were combined and incubated at room temperature for 15 min. Each well was treated with the combined solution for transfection and incubated for 24 h before use in the dextran assay.

### Western blotting

For western blotting of total lysates, HeLa cell lysates were prepared immediately following the indicated treatments by first rinsing with PBS, then into 1× NP40 buffer containing 1X PBS, 20 mM Tris-HCl [pH 7.4], 137 mM NaCl, 1% (v/v), 1%NP40, and 1 mM EDTA) supplemented with EDTA-free protease inhibitor tablets (Roche) and a phosphatase inhibitor cocktail containing sodium fluoride, sodium pyrophosphate, sodium orthovanadate, β-glycerophosphate, and okadaic acid. Samples were sonicated at 40% amplitude in a Q-Sonica Q800R3 water bath sonicator at 30 s ON/30 s OFF intervals for a total of 20 min at 4 °C. Lysates were cleared by centrifugation at 21,000×*g* at 4 °C for 5 min. Protein concentration quantification was analyzed using Pierce BCA Protein Assay Kit (Thermo Fisher Scientific) and samples were diluted to equal concentration using lysis buffer. Lysates were diluted added to 4× Laemmli Sample Buffer (Bio-Rad) supplemented with 10% (v/v) β-mercaptoethanol and boiled for 5 min in a 95 °C prior to SDS-PAGE. SDS-PAGE was performed with the Bio-Rad Mini Protean Tetra Cell gel system with 15% Mini-PROTEAN TGX precast gels at 120V, using Bio-Rad Precision Plus Dual Color standard protein ladder. Electrophoretic transfer to nitrocellulose membranes was performed at 100V for 1 h at 4 °C using a 20% (v/v) ethanol-based transfer solution. Membranes were then stained for protein abundance using Ponceau S solution and rinsed 3 times with 1x TBST (containing 0.1% (v/v) Tween 20 detergent) to remove excess stain. Membranes were blocked in 5% non-fat dry milk solution in 1x TBST for 1 h rat room temperature, rinsed 3 times with 1× TBST, and incubated with primary antibody for 18 h at 4 °C at dilution per manufacturer guidelines. Next, membranes were then washed 3 times with 1× TBST. A 1:5000 dilution of Goat anti-rabbit HRP conjugate or Goat anti-mouse HRP conjugate (GE Healthcare) in a 5% bovine serum albumin 1× TBST solution was used for primary antibody detection of membranes rocking for 1 h at room temperature. Membranes were then washed 3 times with 1× TBST and HRP detection was performed using the SuperSignal West Pico PLUS chemiluminescent substrate. Chemiluminescence was detected and imaged on membranes with the iBright imaging system (Thermo Fisher Scientific). Raw chemiluminescent images were extracted and band intensities quantified using iBright imaging software (Thermo Fisher Scientific; https://www.thermofisher.com/us/en/home/life-science/protein-biology/protein-assays-analysis/western-blotting/detect-proteins-western-blot/western-blot-imaging-analysis/ibright-systems/software.html). Mean gray values of bands at protein size were extracted and plotted using GraphPad Prism 10 (https://www.graphpad.com/updates/prism-10-1-1-release-notes) with the indicated control band used for relative quantification compared to control band. Any brightness adjustments used in figure images were applied equally to the image for improved visualization.

### Immunoprecipitation

For immunoprecipitation, we performed the same cell lysis and protein concentration quantification protocol as used for western blotting described above, then diluted lysates to 1 mg/ml using ice cold wash buffer containing 10 mM Tris-HCL pH 7.2, 5 mM EDTA, 150 mM NaCl and EDTA-free protease inhibitor tablets (Roche), as well as a phosphatase inhibitor cocktail containing sodium fluoride, sodium pyrophosphate, sodium orthovanadate, β-glycerophosphate, and okadaic acid. Then 10 μl of Affi-Prep Protein A magnetic beads (Biorad cat# 156-0006) were equilibrating through washing 3X in 1 ml wash buffer using a magnetic tube rack to pellet between washes. Beads were then resuspended in wash buffer and 3 μg of affinity-purified mab414 antibody was added and rotated for 1 h at room temperature, followed by 3 more washes in wash buffer and removal of final wash using the magnetic tub rack for pellet isolation. Diluted lysate was then added to the equilibrated beads and rotated at 4 degrees overnight. The next day the supernatant was moved to a fresh tube and washed 3X in wash buffer as previously described. The supernatant was discarded and beads were transferred in 50uL wash buffer to a fresh Eppendorf tube where they were suspended in 1X laemmli SDS-sample buffer and boiled for 5 min at 95 °C to dissociate immunocomplexes from beads. Supernatant was then used for SDS-PAGE/Western blot.

## Data availability

Data available upon request from Armen I. Yerevanian ayerevanian@mgh.harvard.edu. Software code DOI for dextran assay analysis will be available after acceptance.

## Supporting information

This article contains supporting information (Supplemental Figs. S1 and S2).

## Conflict of interest

Alexander A. Soukas has financial interests in Atman Health, LLC. Dr Soukas’s interest was reviewed and is managed by MGH and Mass General Brigham in accordance with their conflict-of-interest policies.
