# Supplementary material for: O-GlcNAcylation is a mitochondrial-nuclear signal that regulates passive transport through the nuclear pore complex
Source: J Biol Chem. 2026 Jul 16;302(9):113343. doi: 10.1016/j.jbc.2026.113343 (PMC13488040; doi:10.1016/j.jbc.2026.113343)
Supplement: Supplementary file 1 [file mmc1.docx]

**O-GlcNAcylation is a mitochondrial-nuclear signal that regulates passive transport through the nuclear pore complex**

Talia Hart, Ashley Duke, Yifei Zhou, Alexander A. Soukas, Armen I. Yerevanian

**Material included**

Supplemental Figure S1

Supplemental Figure S2

**Supplemental Figure S1.**

Representative images **(A)** and quantification **(B)** of Panc1 cell nuclei treated with vehicle or 1 mM phenformin for 24 hours, showing passive transport restriction with phenformin treatment via the dextran permeability assay. Cells are incubated with a 40 kD FITC-Dextran, 500 kD TRITC-Dextran, and Hoechst 33342 co-strain. Two independent replicates (n=50-115 nuclei per condition). Graph data points represent individual nuclei and bars indicate mean ± SD.; ∗∗∗∗p < 0.0001; by

Welch’s t-test. Scale bar: 25um.

S-1

S-2

**Supplemental Figure S2.**

**(A)** Representative images of western blot including non-specific IgG immunoprecipitation. IgG control sample was performed with the same input lysate in parallel with the mab414 immunoprecipitation in the experiment in Figure 4C. Representative images **(B)** and quantification **(C)** of FG Nups immunoprecipitated with mAb414 in vehicle or 1 mM phenformin treated HeLa cells and probed with mAb414 and anti-OGlcNAc antibodies. Phenformin treatment shows no discernable changes in O-GlcNAclyation of the other identified FG Nups. **(D)** Western blot probing for total levels of FG Nups using mab414 antibody with actin as a loading control in vehicle or 1 mM phenformin treated HeLa cells indicate no significant change in FG Nup levels with phenformin treatment. **(E)** Gene expression analysis of OGT (left) and OGA (right) in the presence of phenformin through RT-qPCR gene expression analysis normalizing to actin expression, represented as fold changed compared to the control sample (n=3 biological replicates). **(F)** Gene expression analysis confirming knockdown after 24h of treatment using OGT (left) and OGA (right) siRNA represented as fold change in expression in comparison to the negative control siRNA (n=3 technical replicates). Bar graph represents mean ± SD. Data analyzed using a one-way ANOVA followed by Dunnet’s multiple comparison test (C) and by Welch’s t-test (D,E,F) *p< 0.05, ∗∗p < 0.01, ∗∗∗p<0.001, ∗∗∗∗p < 0.0001, ns not significant.

S-2
